# Supplementary material for: Fine Mapping and Candidate Gene Analysis of Pm36, a Wild Emmer-Derived Powdery Mildew Resistance Locus in Durum Wheat
Source: Int J Mol Sci. 2022 Nov 7;23(21):13659. doi: 10.3390/ijms232113659 (PMC9657016; doi:10.3390/ijms232113659)
Supplement: Supplementary file 1 [file ijms-23-13659-s001.zip › Supplemental Figure S1.pdf]

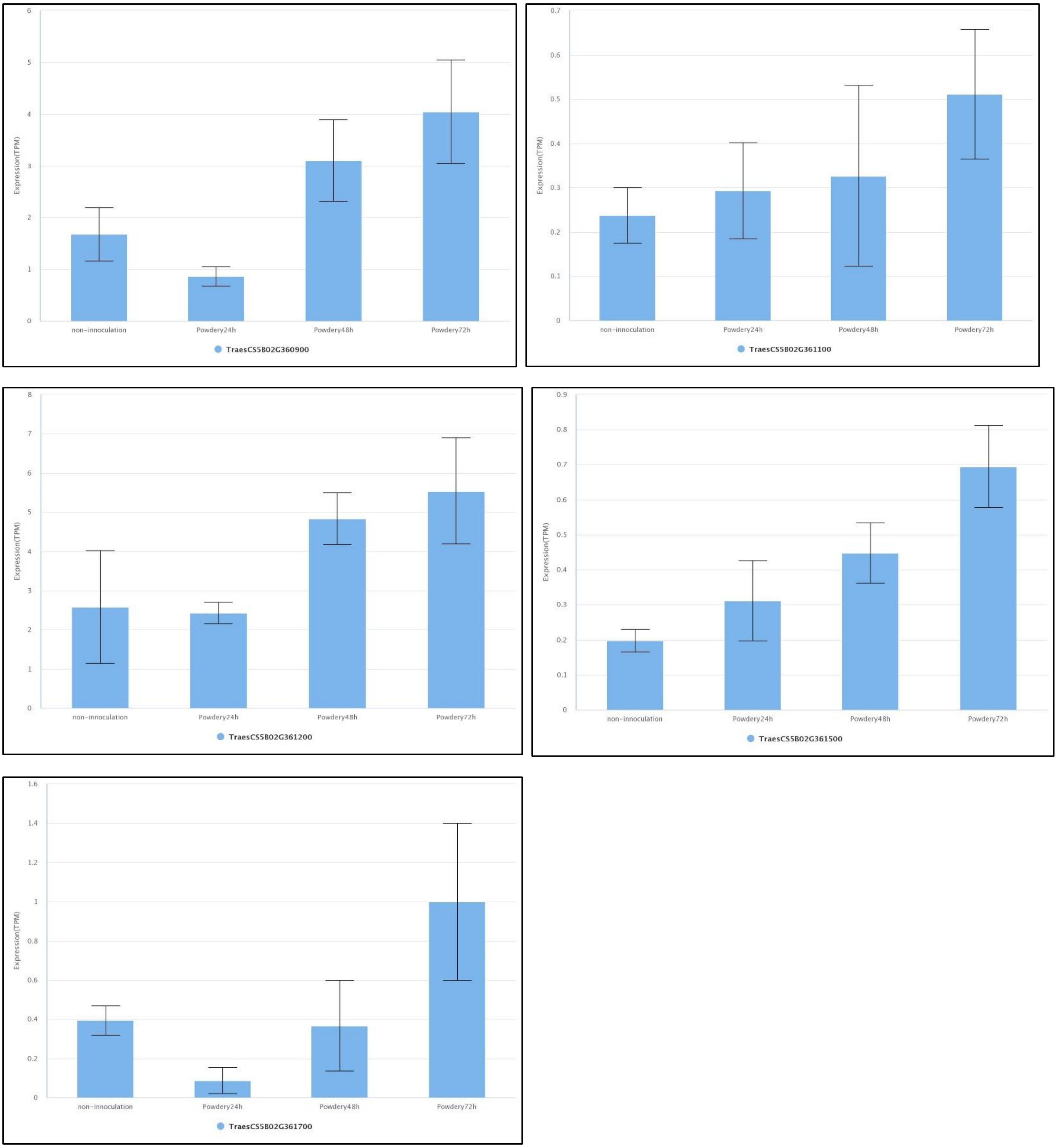


**Figure S1**. In silico expression (TPM) of the bread wheat Chinese Spring genes *TraesCS5B02G360900*, *TraesCS5B02G361100*, *TraesCS5B02G361200*, *TraesCS5B02G361500* and, *TraesCS5B01G361700* on leaves sampled 24, 48 and 72 hours after inoculation [49](http://wheatomics.sdau.edu.cn/).
